# Trans-Influence in Dinuclear Pt(III) Complexes: Electronic Structure, σ‑Donation, and Pt–Pt Spin–Spin Coupling

**DOI:** 10.1021/acs.inorgchem.5c03401

**Published:** 2025-10-17

**Authors:** Pedro P. R. Oliveira, Patrick R. Batista, Lucas C. Ducati, Jochen Autschbach

**Affiliations:** † Department of Fundamental Chemistry, Institute of Chemistry, University of São Paulo, São Paulo, São Paulo 05508-000, Brazil; ‡ Department of Chemistry, University at Buffalo, State University of New York, Buffalo, New York 14260-3000, United States

## Abstract

This study investigates the trans influence in dinuclear platinum­(III) complexes using a combined approach of ab initio molecular dynamics and natural localized molecular orbital (NLMO) analysis. Focusing on pivalamidate-bridged Pt^III^ complexes with axial ligands of varying σ-donation strength, it is quantified how ligand–metal interactions propagate through the Pt–Pt bond, and how they affect bond polarization, axial water coordination, and ^1^ *J*_PtPt_ spin–spin coupling constants. NLMO analysis reveals quantitatively that strong σ-donating ligands polarize the Pt–Pt bond, shifting the electron density toward the opposite platinum center. The polarization mechanism is identified as the primary reason for the observed reduction of ^1^ *J*_PtPt_, because the bond polarization diminishes the transmission of the nuclear magnetic spin-induced electron spin density through the Pt–Pt bond. Additionally, the destabilization of axial water coordination at the opposite Pt site can be rationalized through a polarization-induced Pt^IV^– Pt^II^-like mixed-valence character.

## Graphical abstract

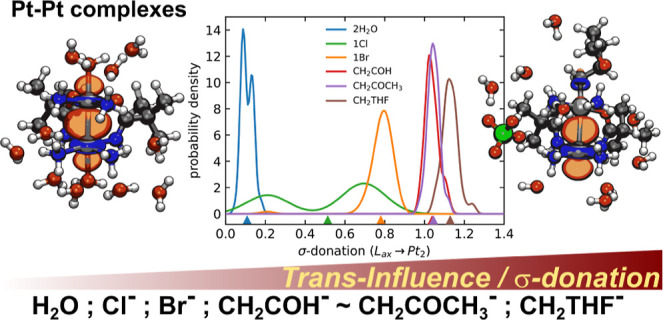

## 1 Introduction

The coordination of a ligand X to a metal ion M in a coordination complex modifies the interactions between the metal and another ligand A in a complex. The classification of the X–M–A moiety as *trans* vs *cis* depends on whether the affected ligand A is adjacent or opposite to ligand X.^1^ Typically, the relevant interactions are described as two distinct but related phenomena,^2–6^ namely, (i) a kinetic effect associated with the change in the rate of substitution of a ligand due to exchange of the trans ligand, and (ii) a structural and energetic influence that takes place via associated modulations of the M–A bonds. Evidently, there must be a corresponding modification of the X–M bond and the associated kinetics by the presence of the M–A coordination, and both are likely also modulated by the presence and type of other ligands, such that it is important to consider the synergy between the different metal–ligand bonds in the system. This work focuses on the trans influence, that is, to what extent and how does the presence of the X–M interaction influence the M–L bond in the trans position. In what follows, the word “effect” is used in its more general sense, not specifically in reference to the aforementioned kinetic effect, unless explicitly stated so.

The trans influence can often be rationalized through σ interactions between ligand X and metal M. A useful way to conceptualize the situation is by representing the bonding system with the following resonance structures, with the relative weights of them in the ground state wave function depending on the nature of the ligands X and A, as well as the metal M:

1 X:⁡⁡M−A↔X−M⁡⁡:A

This resonance scheme illustrates that as the σ interaction X–M strengthens, the contribution of the right-hand resonance form becomes increasingly dominant. Presumably, this enhances the polarization of the M–A bond toward the ligand, while weakening its covalent character.

Since the trans influence modulates the M–A bond, it impacts various properties associated with the electronic structure, including NMR parameters such as the *J*_MA_ coupling constants.^7^ While it is well established, by combining both experimental and theoretical data,^4^ that increasing trans influence tends to reduce the *J*_MA_-coupling, the main underlying reasons should be further investigated in specific systems. Namely, does a reduction of the *J*_MA_ nuclear spin–spin coupling arise primarily from a polarization of the M–A bond, from diminishing spin–spin coupling transmission through the σ-bond (as the resonance scheme suggests), simply via an increased bond distance, or a combination of these factors?

Because they play an important role in the mode of action of Pt^II^-based anticancer drugs,^8^ trans effects have been extensively explored in Pt^II^ complexes.^9^ However, trans effects have not yet been theoretically explored in the less common Pt^III^ complexes, where they involve the metal–metal bond, affecting ligand lability and bond strengths at the opposite metal center. In contrast to Pt­(II) dimers, Pt­(III) complexes are characterized by a metal–metal bond involving two d^7^ platinum atoms with equatorial bridge ligands and axial ligands at both ends of the Pt–Pt axis. The axial ligands play an important role in the chemical properties of the complex in solution. Depending on the ligands and details of the synthesis, Pt­(III) complexes may also assemble into oligomeric chains, including “wires” comprising metal–metal interactions with mixed valences, such as Pt^II^ and Pt^IV^.^10–12^ In this case, one of the platinum atoms adopts a d^8^ electronic configuration interacting with a d^6^ Pt­(IV) center. Such molecular chains have attracted significant interest for their potential applications in light-emitting diodes, photovoltaic cells, and molecular sensors.^12–14^ The present study is concerned with dinuclear Pt­(III) complexes.

Dinuclear Pt^III^ complexes serve as interesting model systems for understanding how the trans influence is transmitted through the Pt^III^–Pt^III^ bond, which can be probed experimentally (and computationally) by ^1^ *J*_PtPt_. Amidate-bridged dinuclear platinum complexes are particularly interesting in this context because the rates of substitution reactions at the axial sites and the associated reaction mechanisms can be strongly influenced by through-bond trans interactions. This, in turn, impacts for instance the efficiency of such complexes as homogeneous catalysts in selective olefin oxidation reactions.^15,16^

Among available quantum chemical methods, the analysis based on localized molecular orbitals (LMOs) appears to be a particularly well-suited tool for the theoretical investigation of these effects. In this approach, delocalized canonical molecular orbitals are transformed into more chemically intuitive localized orbitals representing individual bonds, lone pairs, or core–shells, which can be used to evaluate how electron density is redistributed along the L_1_–Pt–Pt–L_2_ bonding framework. For example, so-called natural LMOs (NLMOs), obtained via the popular natural bond orbital (NBO) algorithms,^17^ have been employed as a basis to decompose ^1^ *J*_PtPt_ coupling constants,^18,19^ enabling identification of the principal orbitals responsible for transmitting the spin–spin coupling and how their contribution changes with the nature of the axial ligand. Furthermore, the natural localized molecular orbital (NLMO) analysis can be used to quantify the extent of σ-donation.^20^

Although NLMO analysis can provide insights into the electronic structure and properties of metal complexes, evaluating fluctuations and correlations between different variables—such as ligand–metal σ-donation, Pt–Pt bond polarization, and ^1^ *J*_PtPt_–as they occur in solution requires configurational sampling. Ab initio molecular dynamics (AIMD) is a powerful tool for this purpose, as it enables simulations of ensemble configurations,^21,22^ while being able to capture bond formation and breaking without the need for a force field with parameters for different metal coordination environments. Furthermore, the trans influence is most commonly characterized via metal–ligand distances in the solid state through X-ray crystallography. However, in solution, solvent interactions can significantly perturb the electronic structure, affecting both Pt–Pt and Pt–ligand bond distances and NMR parameters. AIMD simulations with explicit solvent modeling therefore provide a suitable way to investigate these effects as they occur in solutions.

Motivated by these considerations, we present a combined computational approach using AIMD and NLMO analysis to investigate the trans influence of axial ligands in dinuclear platinum complexes, the propagation of this influence through the Pt^III^–Pt^III^ bond, and its effect on the Pt–Pt *J*-coupling in solution. We analyzed a set of pivalamidate-bridged dinuclear platinum complexes (Figure 1) that covers a range of σ-donation strengths. These complexes have been previously synthesized and experimentally characterized in solution,^23–25^ providing an opportunity for evaluating these effects. Note that we have already shown in previous works^26,27^ that relativistic density functional theory (DFT) calculations of the type used for the present study are capable of reproducing the experimentally observed platinum NMR parameters reasonably well, thus providing a crucial “reality check” of the calculations presented in this work.

**Figure 1:**
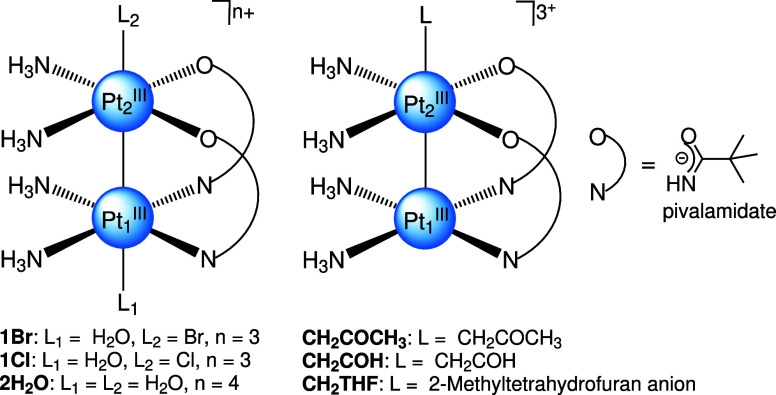
Dinuclear pivalamidate-bridged Pt^III^ complexes investigated in this study. Boldface labels indicate the shorthand nomenclature used in the text. Adapted from *J. Chem. Phys.* 2024, 160, 114307, with the permission of AIP Publishing.

## 2 Computational Methods

AIMD simulations were carried out using the Car–Parrinello (CPMD) approach as implemented in the quantum ESPRESSO program,^28^ version 6.0. The electronic structure was described with the Perdew–Burke–Ernzerhof (PBE) generalized gradient approximation density functional.^29^ Ultrasoft pseudopotentials were used to represent each atom with an effective core potential and smooth pseudo atomic orbital during the simulation. The pseudopotentials employed were obtained from pslibrary version 1.0.0.^30^ The NMR calculations of the spin–spin coupling ^1^ *J*_PtPt_ for 256 sampled AIMD configurations used the PBE0 hybrid functional,^31^ incorporating both scalar relativistic and spin–orbit (SO) effects via the zero-order regular approximation (ZORA) Hamiltonian^32^ as implemented in a 2018 release of the Amsterdam Density Functional (ADF) program.^33–37^ For the Pt atoms, we employed the augmented all-electron Slater-type orbital (STO) basis set for *J*-coupling calculations (jcpl)^38^ from the ADF basis set library, while all other atoms were described by an all-electron STO basis of polarized valence triple-ζ (TZP) quality. This level of theory represents a balance between computational efficiency and accuracy, as is usually the case. Calculated ^1^ *J*_PtPt_ were decomposed into NLMO contributions using a relativistic *J*-coupling analysis developed previously by one of us.^18,19^ NLMOs were generated with the NBO program^17^ (version 6.0) as interfaced with ADF. The same approach was applied to conduct NLMO analysis to assess σ-donation in MD-sampled configurations. To obtain smooth and normalized probability density estimates, we used kernel density estimation^39,40^ with a Gaussian kernel instead of simple histograms. The bandwidth was optimized via Scott’s rule,^41^ as implemented in SciPy Stats^42^ module. The detailed computational protocol applied in this study is described in the Supporting Information. It is based on a combination of AIMD simulations and relativistic KS-DFT NMR calculations, a combination of methods that has been employed also in our previous work.^26,27,43,44^

A brief comment on the expected accuracy of the electronic structure model is in order. Namely, there is ample demonstration in the literature that heavy-element NMR chemical shift and *J*-coupling calculations can be as accurate with ZORA as they are with fully relativistic methods,^45–51^ especially when taking into account other approximations—e.g., the DFT functional and basis set—that are used in such calculations. (We note in passing, however, that ZORA is not sufficiently accurate for predicting the absolute shielding of heavy isotopes because of deep-core contributions to the shielding; however, those cancel in the chemical shift.^45,48^ ) The combination of ZORA/PBE0 with the chosen STO basis sets was previously benchmarked for *J*-coupling involving third–row transition metals^38^ and gave approximately 8% median unsigned deviation with respect to experimental data for coupling constants involving one Pt center, and about 12% median unsigned deviation for a larger set including W, Pt, Hg, Tl, and Pb.

## 3 Results and Discussions

### 3.1 Trans Influence and NLMO Analysis

With the goal of evaluating the variation in L–Pt σ-donation across different ligands during the dynamics, we conducted NLMO analyses on configurations sampled from the AIMD simulations. The analysis generates localized NBOs designed to resemble a classical Lewis structure, including bonding, nonbonding, and lone pair (LP) orbitals, based on internal program thresholds.^52^ The NLMOs are generated as linear combinations of NBOs, capturing electron delocalization when they are present. When localization is strong, NLMOs closely resemble their “parent” NBOs. Conversely, when electron delocalization is important, the NLMOs exhibit non-Lewis character, with parent NBO occupancies less than 2 (see further details in the Supporting Information, Section S1). For each sampled configuration, the extent of σ-donation was quantified as the metal density weight in the occupied L–Pt orbital, regardless of its NBO classification type (delocalized LP vs bond). Figure 2 presents the resulting probability density functions (PDFs) of the extent of σ-donation in the AIMD simulations, along with the corresponding expectation values (AIMD averages; see Section S2 in the Supporting Information for additional information).

**Figure 2:**
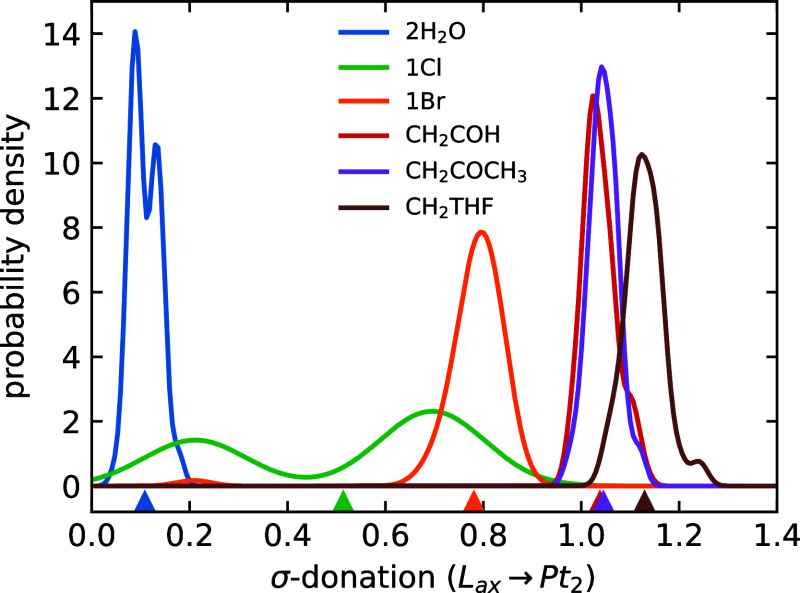
Normalized PDFs for the extent of σ-donation (L_ax_ → Pt_2_) from NLMO analysis of 64 AIMD-sampled configurations. Triangles indicate the overall AIMD averages.

The observed trend in σ-donation strength follows the order

$$H_{2}O<{Cl}^{-}<{Br}^{-}<{CH}_{2}{COH}^{-}∼{CH}_{2}{{COCH}_{3}}^{-}<{CH}_{2}{THF}^{-}$$

which is consistent with qualitative considerations: water ligands exhibit weaker donation compared to halides (Cl, Br), while the covalently bound carbon-based ligands show the strongest σ-donation as expected.^53^

An interesting behavior is observed for the chloro ligand, which shows two distinct peaks in the PDF image in Figure 2. This bimodal distribution reveals the existence of two well-defined bonding regimes: those dominated by LP character (with σ-donation values around 0.2) and those with pronounced bonding character (showing σ-donation values around 0.7). A visual comparison of representative σ-donating NLMOs from the two regimes is shown in Figure S5. This clear separation allows the configuration space to be partitioned into two events—LP and bonding—enabling the estimation of conditional expectation values. Notably, a strong correlation between the σ-donation strength and the Cl–Pt_2_ distance was observed. The LP regime corresponds to a larger average distance of 2.48 °A, while the bonding regime has an average distance of 2.39 °A (Figure S6). This average contraction of 0.1 °A in the bond distance reflects the increased covalent character of the metal–ligand interaction and provides a structural evidence that supports the classification of this complex into two distinct bonding regimes in solution.

The potential for π-interactions in these platinum halide complexes was also investigated. To measure π-donation from the halide p orbitals to the metal, we quantified the metal orbital contributions to the halide LP NLMOs (see Section S3.6). Our analysis shows that π-donation is exceedingly weak, with halide atomic orbitals contributing over 99% of the density of the relevant LP NLMOs. This confirms that the bonding is overwhelmingly dominated by σ-interactions, with no significant π-donation from halide ligands. Furthermore, as expected, back-donation from platinum nonbonding orbitals is also insignificant, as shown in Figure S8. Back donation is typically observed with ligands different than those studied herein, such as CO, NO, or CN^–^, which possess low-energy, unoccupied π* orbitals able to accept density from the metal.^53^

Concerning the organic axial ligands, it is noteworthy that the extent of donation is sensitive to the chemical environment and hybridization of the carbon atom bound to the metal-coordinating carbon. Namely, the CH_2_THF ligand donates about 0.1 electrons more than the other two studied carbon-coordinating ligands. The weaker donation by the latter ligands can be attributed to the electron-withdrawing effects within the carbonyl groups. As shown later, this has an effect on the solvent–water interaction at the formally vacant Pt_1_ site of these complexes.

As suggested by the notion of trans influence, axial L–Pt coordination should have a concomitant effect on the Pt–Pt bond. This is corroborated by the distribution of the Pt–Pt σ-bonding orbital over the two Pt centers. Table 1 lists the density weight percentages of the relevant NLMO on the two Pt centers, averaged over the AIMD trajectories as a function of the axial ligand. The trends seen in the table are rather suggestive: The stronger the donation for a given ligand (Figure 2), the longer is the Pt–Pt distance and the less equal is the Pt–Pt bonding orbital shared between the two Pt centers. For the stronger donating ligands, the Pt_2_–Pt_1_ bond polarizes more strongly toward Pt_1_. In other words, for L = H_2_O or Cl, we have a description corresponding to L: Pt_2_–Pt_1_, whereas for the stronger donating/more covalently bound ligands, the description changes to L–Pt_2_: Pt_1_. This situation is exemplified in Figure 3, which shows the Pt–Pt NLMO of representative AIMD snapshots for the **2H**_**2**_ **O** vs the **CH**_**2**_ **THF** complex. The figure provides a compelling visual representation of the trans influence. It is worth noting that the changing description of the bond pattern reflects an increase in the mixed-valence Pt_2_^IV^–Pt_1_^II^ character, which is also suggested by concomitant changes in the experimental ^195^Pt chemical shifts as reported by Matsumoto and co-workers.^54^ This analysis provides a conceptual framework for understanding the reduced electrophilicity at Pt_1_ in these compounds, which has important implications for water coordination, as discussed next.

**Table 1:** AIMD-Averaged Pt–Pt Distances (Å) and Corresponding Density Weight-% of the Main Pt–Pt Bonding NLMO on Each Pt Center, Computed from 64 AIMD Configurations

ligand L or L_2_	*d*_PtPt_	Pt_2_	Pt_1_
H_2_O	2.60	48	49
Cl	2.62	33	53
Br	2.63	22	56
CH_2_COCH_3_	2.72	10	81
CH_2_COH	2.72	10	82
CH_2_THF	2.77	5	88

**Figure 3:**
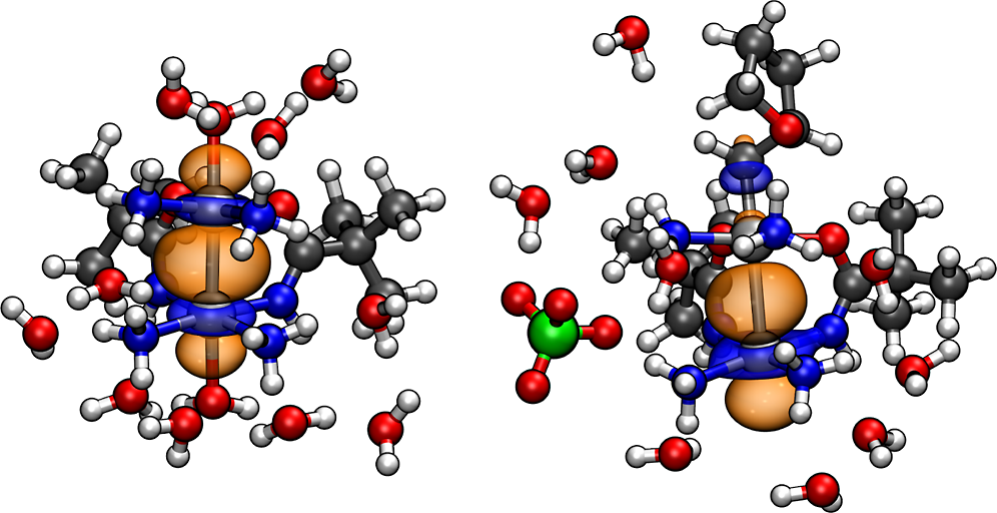
Depiction of the Pt–Pt σ-bonding NLMO (±0.03 isosurfaces) for the axial ligand L (at the top in each image) = **2H**_**2**_ **O** (weakly σ-donating ligand, left graphic) vs L = **CH**_**2**_ **THF** (strongly σ-donating ligand, right graphic).

### 3.2 Trans Influence and Axial Ligand Coordination

Figure 4 presents the Pt_1_–O radial distribution functions (RDFs) for the complexes (left) along with the corresponding coordination numbers calculated from a differentiable switching function (right). This analysis quantitatively addresses how the axial ligand σ-donation strength at the Pt_2_ site affects water coordination at the Pt_1_ site.

**Figure 4:**
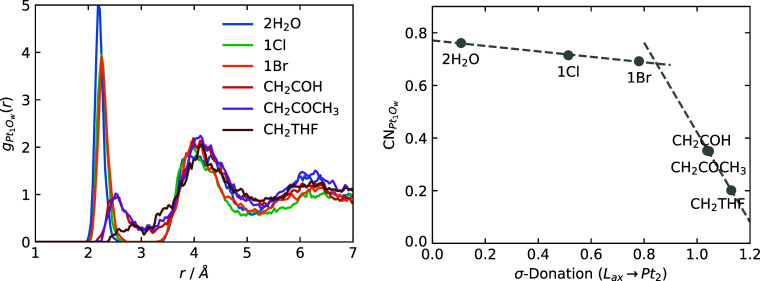
Left: RDFs of the Pt_1_ site and water oxygen atoms. Right: average coordination number (CN) between the Pt_1_ site and water O atoms vs the σ-donation for the different axial ligands at the Pt_2_ site.

The RDFs reveal distinct coordination behavior: Complexes with weaker σ-donating ligands (**2H**_**2**_ **O**, **1Cl**, and **1Br**) exhibit stable water coordination at Pt_1_, as evidenced by a sharp peak at *r* ∼ 2.1 Å that is distinct from the first solvation shell at *r* ∼ 4.1 \Angstrom. In contrast, complexes with carbon-bound ligands present only broad peaks at *r* ∼ 2.4 Å, suggesting at best weak and transient Pt_1_–O coordination bond formation. This effect is most pronounced for the **CH**_**2**_ **THF** complex, which shows no discernible peak for axial water coordination at the Pt_1_ site. The coordination number plot (Figure 4, right) shows a marked change around a σ-donation strength of 0.85, marking a transition from a relatively stable water ligand at the Pt_1_ site to weak and transient water binding. The two panels in Figure 4 go hand-in-hand and are easily rationalized by the data in Table 1: A buildup of electron density on the Pt_1_ site, resulting from the polarization of the Pt–Pt bond toward Pt_1_ for the strongly donating ligands L at the Pt_2_ site, disfavors Pt_1_–O­(water) coordination. We might call this a trans–trans influence. Despite the increase of electron density at the Pt_1_ site, however, the AIMD data showed no compelling evidence for “inverse hydration”, i.e., coordination by water hydrogens.

Extending this analysis, one can infer, based on the trans-ligand σ-donation strength, that the **3Cl** and **3Br** pivalamidate-bridged complexes (see the inset in Figure 5, left) in solution ought to maintain a water molecule coordinated at the vacant Pt_1_ site. This prediction is indeed supported by the MD simulations, which show a sharp peak at *r* ∼ 2.1 Å in the Pt_1_–O_w_ RDFs that integrates to 1.0. Further support comes from Pt chemical shift calculations of the optimized structures (Figure 5, right), which show better agreement with the experimental solution-phase data when a coordinated water molecule is included.

**Figure 5:**
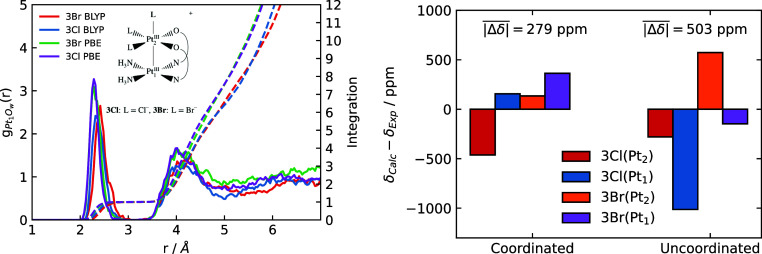
Left: RDFs of the Pt_1_ site and water O atoms for the L = halide complexes shown in the inset. AIMD with PBE and BLYP functionals are compared. Right: deviation between calculated Pt_1_ and Pt_2_ chemical shifts (optimized structures at PBE0/ZORA-SO/TZP­(jcpl) level) with and without water coordination at Pt_1_ site, compared to experimental values. The water-coordinated species exhibits, on average, closer values to experiment in the static calculations, as indicated by the lower mean absolute deviation, $\overset{®}{\left|\Delta \delta \right|}=\overset{®}{\left|\delta_{Calc}-\delta_{\exp}\right|}$ . This is consistent with the simulations, where water binding occurs and the uncoordinated species is not sampled.

The average contributions of Pt_2_ and Pt_1_ to the Pt–Pt NLMO were calculated to be 31% and 55%, respectively, for **3Cl** and 21% and 58%, respectively, for the **3Br** complex. These values indicate similar, if slightly stronger, Pt–Pt bond polarization compared to **1Cl** and **1Br**, respectively (Table 1). Our calculations suggest that while equatorial halide ligands have an influence on the Pt–Pt bond polarization, the effect is not strong enough to induce a Pt^IV^–Pt^II^ type polarization that could leave the Pt_1_ center uncoordinated. Matsumoto and co-workers synthesized the compounds in solution but were unable to isolate them, confirming their formation by ESI MS based on the mass-to-charge ratio.^55^ Our findings indicate that in solution complexes **3Cl** and **3Br** are characterized by the presence of a coordinated water molecule at the Pt_1_ center.

### 3.3 Trans Influence on ^1^ *J*_PtPt_

The experimental characterization of dinuclear Pt^III^ complexes in solution is a challenging task, but the complexes also present considerable challenges for computational studies. For instance, the ^1^ *J*_PtPt_ coupling constant is highly sensitive to the electronic structure, geometrical parameters, and the treatment of solvent and solvent–solute dynamics. In the MD, this leads to considerable ensemble fluctuations Δ*J*, making it a very challenging property for theoretical calculations, as it highlights the limitations of static theoretical treatments for these systems. Please note that we forego comparisons of the MD-averaged ^1^ *J*_PtPt_ to experimental data, as this was already done in ref 27, and focus instead on the trans influence context.

Figure 6 presents the estimated PDFs of ^1^ *J*_PtPt_ along with their ensemble averages for the studied complexes, derived from the AIMD simulations and subsequent relativistic NMR calculations. It can be noted that the *J*-coupling averages follow the expected trend, namely, a lengthening and polarization of the Pt–Pt bond reduces the ensemble-averaged *J*-coupling. It can also be noted that the complexes with smaller ^1^ *J*_PtPt_ have much sharper distributions of ^1^ *J*_PtPt_ values. This seemed to us counterintuitive at first, because the systems with larger Pt–Pt distances, more polarized Pt–Pt bonds, and concomitant smaller ^1^ *J*_PtPt_ also sample a larger range of Pt–Pt distances, as shown below. This would typically be expected to lead to larger, not smaller, fluctuations in the coupling constant. Fortunately, this effect can be rationalized based on the NLMO analysis of ^1^ *J*_PtPt_.

**Figure 6:**
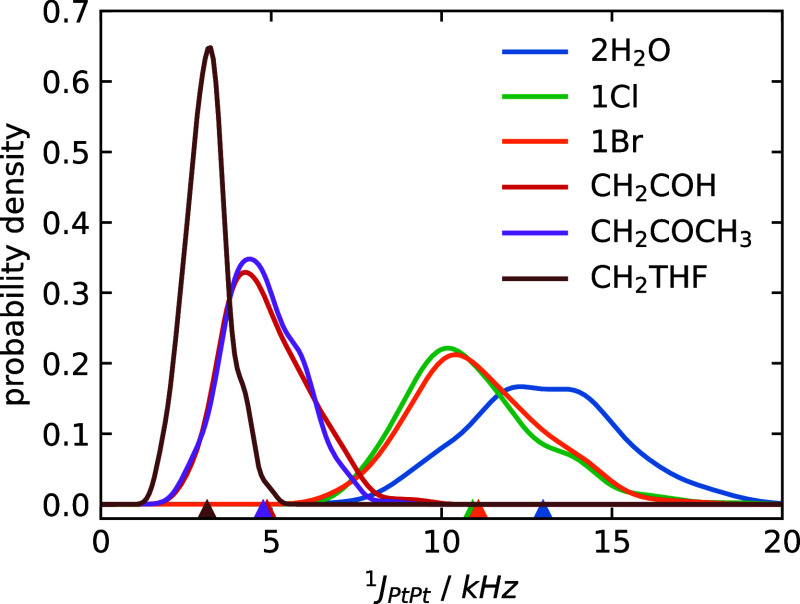
Normalized ^1^ *J*_PtPt_ PDFs obtained by sampling 256 configurations from AIMD simulations. The NMR calculations were performed with PBE0/ZORA-SO/TZP­(jcpl). Triangles indicate the overall averages.

Figure 7 (left) shows the decomposition of ^1^ *J*_PtPt_ for a representative configuration obtained from each MD simulation. For the ligands with strong σ-donation, the contribution of the Pt–Pt bond orbital to the coupling constant tends to be less important. This trend must be connected to the enhanced polarization of the Pt–Pt bond, such that the diminished covalency of the Pt–Pt bond reduces the transmission of the *J*-coupling through this orbital. As a result, both the total ^1^ *J*_PtPt_ value and the dominant orbital contribution (from the Pt–Pt bonding orbital) decrease. The left panel of Figure 7 also indicates that for those systems with a strongly donating axial ligand and strong Pt–Pt bond polarization, most of ^1^ *J*_PtPt_ is transmitted by other orbitals, which are presumably less sensitive to the Pt–Pt distance fluctuations. This is likely the reason for the situation illustrated in the right panel of Figure 7: Even though there are large Pt–Pt distance fluctuations for the **CH**_**2**_ **THF** complex, ^1^ *J*_PtPt_ varies much less than it does for the **2H**_**2**_ **O** complex because the σ­(Pt–Pt) orbital is not the dominant contributor, as shown in Figure S9. As it was previously addressed for pyridonate-bridged complex derivatives^26^ and also observed here, ^1^ *J*_PtPt_ in these Pt­(III) dinuclear compounds is dominated by the relativistic analog of the Fermi Contact (FC) mechanism, as it is often the case for NMR *J*-coupling. Furthermore, the **2H**_**2**_ **O**, **1Cl**, and **1Br** complexes exhibit a stronger influence of the Pt *s*-character in the Pt–Pt bond, which is directly related to the FC mechanism. As the Pt–Pt distance increases, the axial ligand–Pt distance tends to shorten, and the *s*-character increases, yielding higher ^1^ *J*_PtPt_ values. On the other hand, the Pt–Pt distance tends to shorten when the axial ligand–Pt distance increases. This indirect relationship between Pt–Pt distance and ^1^ *J*_PtPt_ also explains why the variations of ^1^ *J*_PtPt_ for the **2H**_**2**_ **O** complex are much stronger in comparison.

**Figure 7:**
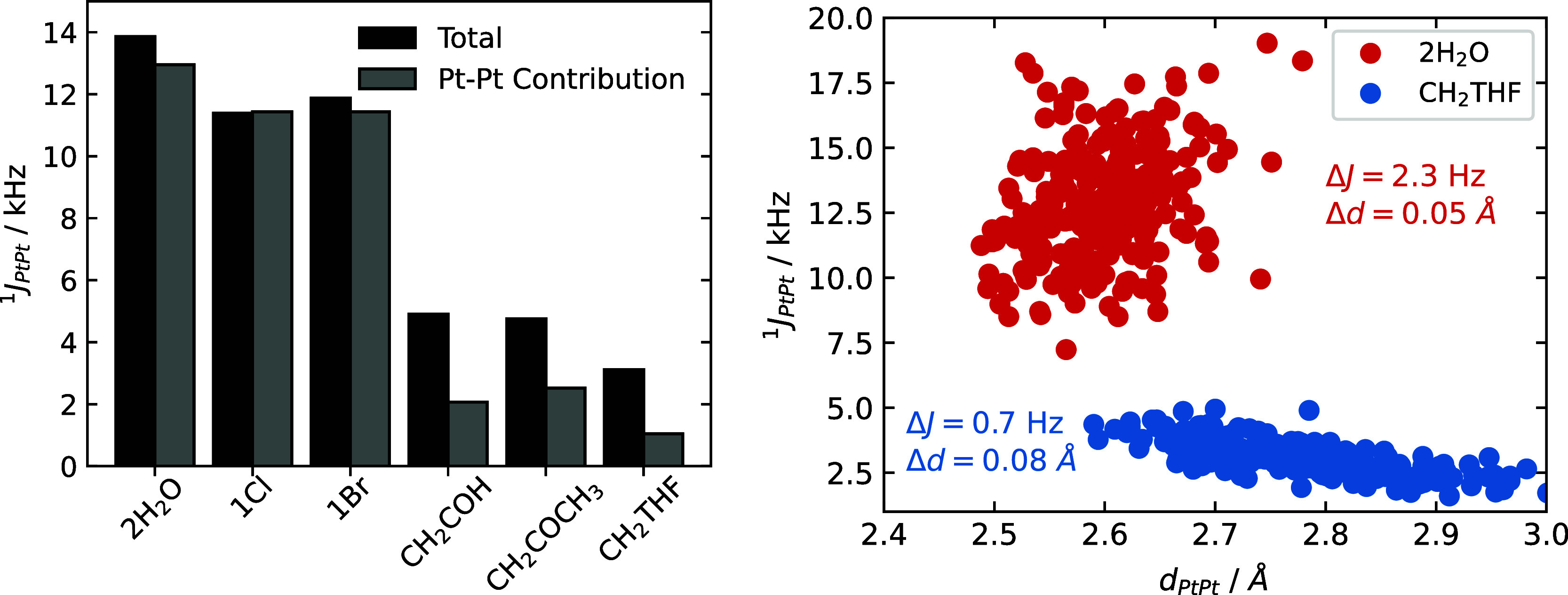
Left: ^1^ *J*_PtPt_-coupling total value and the contribution from only the Pt–Pt bonding orbital (NLMO) for representative configurations obtained from MD the simulations. The configurations were chosen based on having ^1^ *J*_PtPt_ being close to the AIMD-average value. Right: ^1^ *J*_PtPt_ vs *d*_PtPt_ for 256 AIMD-sampled configurations of **2H**_**2**_ **O** and **CH**_**2**_ **THF** complexes.

## 4 Conclusions

AIMD combined with NLMO analysis has proven to be a useful approach for investigating the trans influence in dinuclear platinum­(III) complexes. In this work, the σ-donation strength of different trans ligands in pivalamidate-bridged Pt^III^ complexes has been systematically evaluated, providing not only a quantitative measure of σ-donor effects but also insights into the dynamic ensemble fluctuations. The resonance scheme (eq 1) served as a conceptual framework for rationalizing trends in electrophilicity and ^1^ *J*_PtPt_ coupling constants.

The combined approach revealed that strong σ-donating ligands induce strong polarization of the Pt–Pt bond, shifting the electron density toward the opposing platinum center. This effect leads to a transition from a covalent Pt–Pt bond to a mixed-valence Pt^IV^–Pt^II^-like state as σ-donation increases. The trans influence is transmitted to the opposite platinum site (trans–trans), modulating the stability of axial water coordination through this polarization mechanism. Weak σ-donors (e.g., H_2_O, Cl^–^, and Br^–^) allow for stable water binding, while strong σ-donors (e.g., CH_2_THF^–^) reduce the electrophilicity of the Pt_1_ center, disfavoring axial coordination.

The ^1^ *J*_PtPt_ spin–spin coupling constant decreases with increasing axial σ-donation due to reduced electron sharing across the Pt–Pt bond, which reduces both the magnitude and relative contribution of the σ­(Pt–Pt) orbital to the coupling. Counterintuitively, fluctuations in ^1^ *J*_PtPt_ diminish as the bond weakens, which highlights the dominant role of bond polarization over distance variability in explaining ^1^ *J*_PtPt_ trends in these dinuclear platinum complexes.

## Supplementary Material



## Data Availability

The XYZ coordinates from AIMD snapshots underlying this study are available at 10.5281/zenodo.17078535.

## Notes

Includes details about temperature fluctuations of the Car–Parrinello Molecular Dynamics simulations, solvent structure, convergence of the evaluated sampled variables (^1^ *J*_PtPt_ and σ-donation), π-donation and π-back-donation analysis, and NLMO decomposition of ^1^ *J*_PtPt_-coupling; additionally, it provides theoretical background on estimating ensemble averages and distribution functions from MD simulations (PDF)
