# Supplementary material for: Trans-Influence in Dinuclear Pt(III) Complexes: Electronic Structure, σ‑Donation, and Pt–Pt Spin–Spin Coupling
Source: Inorg Chem. 2025 Oct 17;64(43):21501–8. doi: 10.1021/acs.inorgchem.5c03401 (PMC12587392; doi:10.1021/acs.inorgchem.5c03401)
Supplement: Supplementary file 1 [file ic5c03401_si_001.pdf]

Supporting Information for:

Trans-Influence in Dinuclear Pt(III) Complexes: Electronic Structure, σ-Donation, and Pt–Pt Spin-Spin Coupling

Pedro P. R. Oliveira[a], Patrick R. Batista[a,b], Lucas C. Ducati[a,*],
Jochen Autschbach[c,*]

[a]Department of Fundamental Chemistry
Institute of Chemistry
University of Sao Paulo
São Paulo, SP, 05508-000, BR
email: patrick@iq.usp.br
email: ducati@iq.usp.br

[b]Institute of Chemistry
University of Campinas
Campinas, SP, 13083-862, BR

[c]Department of Chemistry
University at Buffalo
State University of New York
Buffalo, NY 14260-3000, USA
email: jochena@buffalo.edu

September 28, 2025

Contents

S1 Computational Details **S3**

S2 Theoretical Background for Estimating the σ-donation **S4**
S2.1 Probability Density Functions in the Canonical Ensemble S4
S2.2 Point Estimation from MD Simulations S4
S2.3 Histograms and Kernel Density Estimation S5

S3 Complementary Material **S7**
S3.1 Assessment of the CPMD Simulations S7
S3.2 Solvent Structure S8

S3.3 Coordination Number Calculation S9
S3.4 Convergence of $^1J_{\mathrm{PtPt}}$ S9
S3.5 Convergence of σ-Donation S10
S3.6 π-donation and π-backdonation S13
S3.7 NLMO Decomposition of $^1J_{\mathrm{PtPt}}$ S14

S1 Computational Details

Molecular structures of the complexes, previously optimized with the hybrid PBE0 functional, were arranged in a cubic cell containing 64 solvent molecules (water plus counterions) using the PACKMOL program[1]. The cell dimensions were chosen to reproduce the density of heavy water under ambient conditions. Since the experimental samples for obtaining the NMR spectra of complexes were prepared using an acidic solution of D_2O ($DClO_4/D_2O$), perchlorate anions (ClO_4^-) were added based on the charge of each complex to maintain cell charge neutrality in a total of 65 molecules (the solute, water molecules, and appropriate number of perchlorate anions). All hydrogen atoms were replaced with deuterium also to aid in the adiabatic separation of electronic and nuclear degrees of freedom. Prior to the simulations, the initial system (solute + solvent) underwent a restricted optimization with a universal force field with the solute constrained, in order to provide a more suitable initial geometry, thereby facilitating the initial determination of the Kohn-Sham orbitals.

Car-Parrinello Molecular Dynamics (CPMD) simulations were performed with a kinetic energy cutoff of 100 Ry, a fictitious electron mass of 450 a.u., and a time step of 5.0 a.u. (equivalent to 0.12 fs). The only available Grimme's dispersion correction (D2)[2] in QuantumEspresso (QE) program, CP module, at this time was included to improve the description of intermolecular interactions. After the initial 'wave function optimization' (in QE parlance), the molecular dynamics simulations were conducted in two stages: thermalization and production. Thermalization was performed in the canonical (NVT) ensemble using the Nosé-Hoover thermostat[3] at an elevated temperature of 350 K to mitigate over-structuring of the solution. The over-structuring is a known issue in *ab initio* simulations of water with PBE and other GGA functionals, and the use of an elevated temperature is a commonly used remedy.[4] The systems were thermalized until thermal equilibrium was reached for about 3 ps. Subsequently, the production run of the CPMD was carried out in the microcanonical (NVE) ensemble for 30 ps. The ions and electrons were propagated using the Velocity-Verlet algorithm, and the time evolution of atomic coordinates was recorded and stored every 10 steps (1.2 fs) as trajectories for subsequent structural analysis. The CPMD simulations were analyzed using the TRAVIS software[5,6] by data such as radial distribution functions (RDFs), and time evolution and average of distances and angles. Trajectory visualization was performed with the VMD[7].

To calculate ensemble averages of the σ-donation values and $^1J_{\mathrm{PtPt}}$ spin-spin coupling constants we sampled 64 and 256 uniformly spaced configurations from the NVE trajectory, corresponding to time intervals of 0.45 ps and 0.11 ps, respectively. These computations employed a microsolvation approach, where solvent molecules were selected based on their proximity to the solute. This was determined by ranking all intermolecular solute-solvent contacts by increasing distance. The procedure consistently identified the 10 closest solvent molecules as sufficient for achieving convergence in the explicit solvent effect on $^1J_{\mathrm{PtPt}}$ values.[8] Solvent effects were globally addressed using explicit solvent molecules (microsolvation) for short-range interactions combined with the conductor-like screening model (COSMO[9], using the permittivity of 78.8 to represent the water as solvent), for simulating the solvent bulk effect.

The *J*-coupling analysis was performed with the NBO program[10] (version 6.0) as interfaced with ADF, using the PBE0[11] hybrid functional and employing the jcpl[12] basis set for Pt atoms and TZP basis set for all other atoms. The Self-Consistent Field threshold and Becke[13] integration grid for this calculations were set to 1×10^{-6} and "verygood", respectively. The representative trajectory configurations were used, which presented $^1J_{\mathrm{PtPt}}$ closer to the total average. To apply this approach to relativistic NMR calculations, a set of localized orbitals (NBOs and NLMOs) is derived from the scalar relativistic (SR) electronic structure, while the property calculation includes all relevant spin-orbit (SO) coupling terms. The connection between the two sets of orbitals is established by projecting the SO

orbitals onto the complete set of SR orbitals. The sum of all terms in this analysis reproduces the NMR parameters obtained from variational SO-DFT. This allows for a chemically intuitive decomposition of $^1J_{\mathrm{PtPt}}$ in terms of SR localized orbitals.

S2 Theoretical Background for Estimating the σ-donation

In the main work, distributions of σ-donation and $^1J_{PtPt}$-coupling constants have been estimated. From a mathematical point of view, these quantities are well defined for any configuration and can be treated as continuous random variables over the configurational space. This section provides a brief overview of the methodology used to derive these estimates from molecular dynamics simulations. While we illustrate the basic equations in the canonical ensemble, they can be readily modified to describe other ensembles by substituting the appropriate probability density functions.[14]

S2.1 Probability Density Functions in the Canonical Ensemble

One of the corner-stone results from classical statistical mechanics is that for any macroscopic system in thermodynamic equilibrium with a fixed number of particles N, volume V and temperature T, the probability density function over the configurational space Σ_X is a *Boltzmann distribution* on the potential energy $U(\mathbf{x})$ of the configuration $\mathbf{x} \equiv (\mathbf{x}_1, \mathbf{x}_2, \cdots, \mathbf{x}_N) \in D(V) \subset \Sigma_X$,

$$f(\mathbf{x}) = \frac{1}{Z}\mathrm{e}^{-\beta U(\mathbf{x})} \quad \text{with} \quad Z = \int_{D(V)} \mathrm{e}^{-\beta U(\mathbf{x})} d\mathbf{x}. \tag{S1}$$

In Equation S1, Z is a normalization constant called *configurational partition function*, $\beta = 1/kT$ (where k is Boltzmann's constant) and $D(V)$ is the accessible region of the configurational space, imposed by the system finite size constraint. From this result it is, in principle, straightforward to calculate the expectation value for any property $A = A(\mathbf{x})$, which depends only on the atomic positions $\mathbf{x}$, by integrating over the entire accessible configurational space:

$$\langle A(\mathbf{x}) \rangle = \frac{1}{Z} \int_{D(V)} A(\mathbf{x}) \mathrm{e}^{-\beta U(\mathbf{x})} d\mathbf{x}. \tag{S2}$$

From a mathematical standpoint, the quantity $A : \Sigma_X \to \mathbb{R}$ is a random variable on the configurational space. It is possible, then, to write the *probability density function (PDF) of A* by grouping together the probability densities of all the degenerate configurations in a, $\forall\, a \in \mathbb{R}$:

$$f_A(a) = \frac{1}{Z} \int_{D(V)} \delta(A(\mathbf{x}) - a) \mathrm{e}^{-\beta U(\mathbf{x})} d\mathbf{x}, \tag{S3}$$

where the Dirac delta function restricts the integration to configurations in which $A(\mathbf{x}) = a$. From the PDF of A, the associated expectation value $\langle A \rangle$ and variance $\left\langle (\delta A)^2 \right\rangle$ can be computed directly as

$$\langle A \rangle = \int_{-\infty}^{\infty} a f_A(a) da \quad \text{and} \quad \left\langle (\delta A)^2 \right\rangle = \int_{-\infty}^{\infty} (a - \langle A \rangle)^2 f_A(a) da. \tag{S4}$$

S2.2 Point Estimation from MD Simulations

The expectation value of any quantity $A : \Sigma_X \to \mathbb{R}$ of interest can theoretically be calculated using Equation S2 or by knowing S3. In practice, however, solving the $3N$ integrals for a realistic potential in

a complex atomistic system is computationally unfeasible. In the so called *Molecular Dynamics* (MD) approaches, system configurations are generated via a *dynamical system*. Based on the *ergodic hypothesis*, the time-averaged value of A approximates the ensemble average and

$$\langle A \rangle = \lim_{\tau \to \infty} \frac{1}{\tau} \int_0^\tau A(\mathbf{x}(t))dt, \tag{S5}$$

where $A(\mathbf{x}(t))$ is evaluated and integrated along the entire trajectory $\mathbf{x}(t)$ in configurational space. Since the equations of motion in practice can only be solved in discrete time steps, the continuous trajectory is approximated using $\mathcal{N}$ steps of size Δt, with $\tau = \mathcal{N}\Delta t$. Therefore, an *estimate* for the integral is

$$\frac{1}{\tau} \int_0^\tau A(\mathbf{x}(t))dt \approx \frac{1}{\mathcal{N}} \sum_{k=1}^{\mathcal{N}} A_k \equiv \overline{A} \tag{S6}$$

where $A_k = A(\mathbf{x}((k-1)\Delta t))$. Equation S6 represents a *sample average* of $\mathcal{N} \gg 1$ samples from the distribution f_A (Equation S3). Since A_k are identically distributed random variables, the sample average is also a random variable and a *unbiased* estimator for the expectation value of the sampled distribution since:

$$\left\langle \overline{A} \right\rangle = \left\langle \frac{1}{\mathcal{N}} \sum_{k=1}^{\mathcal{N}} A_k \right\rangle = \frac{1}{\mathcal{N}} \sum_{k=1}^{\mathcal{N}} \langle A_k \rangle = \langle A \rangle . \tag{S7}$$

If only *uncorrelated* configurations from the trajectory are considered, it is assumed that the A_k values will then be *independent and identically distributed RVs (i.i.d.)*. It is possible to show that the variance of $\overline{A}$ will be given by[15]:

$$\left\langle (\delta \overline{A})^2 \right\rangle = \frac{\left\langle (\delta A)^2 \right\rangle}{\mathcal{N}}. \tag{S8}$$

Therefore, when a sample mean is computed from more configurations it becomes a more *precise estimator* of the underlying distribution's expectation. In fact, by Chebyshev's inequality, a random variable X with finite expectation and variance satisfies

$$\mathbb{P}\left(|X - \langle X \rangle| > \epsilon\right) \le \frac{\left\langle (\delta X)^2 \right\rangle}{\epsilon^2}, \tag{S9}$$

for any $\epsilon > 0$. Therefore, the sample average of independent samples of f_A *converges in probability* to the expectation of A:

$$\lim_{\mathcal{N} \to \infty} \mathbb{P}\left(\left| \overline{A} - \langle A \rangle \right| \ge \epsilon \right) = 0. \tag{S10}$$

We then say that the sample average is a *consistent* estimator of the expectation value. The notation $\overline{A} \xrightarrow{P} \langle A \rangle$ is also common.

S2.3 Histograms and Kernel Density Estimation

A *histogram* can be constructed to estimate the PDF of a random variable f_A from sampling a unbiased molecular dynamics simulation. We will show that a histogram converges in probability to the underlying

sampling distribution. To see this, let $\{A_k\}_{k=1}^{n}$ be i.i.d. samples drawn from f_A. If we discretize the image of A in *bins* with a *bin width* of Δa, the probability of A being in the bin centered in a, for small Δa, can be calculated by:

$$\mathbb{P}(A \in [a - \Delta a/2, a + \Delta a/2]) \equiv p \approx f_A(a)\Delta a. \tag{S11}$$

Define the following auxiliary variable:

$$H(a) = \begin{cases} 1/\Delta a & \text{if } A \in [a - \Delta a/2, a + \Delta a/2] \\ 0 & \text{if } A \notin [a - \Delta a/2, a + \Delta a/2]. \end{cases} \tag{S12}$$

The expectation of $H(a)$ will then be:

$$\begin{aligned} \langle H(a) \rangle &= \frac{1}{\Delta a} \cdot p + 0 \cdot (1 - p) \\ &\approx f_A(a). \end{aligned} \tag{S13}$$

Therefore, the sample average of $H(a)$, which in just the number of samples in the bin $[a \pm \Delta a/2]$ (denoted by n_a) divided by the total number of samples $\mathcal{N}$ and the bin width Δa,

$$\overline{H}(a) = \frac{1}{\mathcal{N}} \sum_{k=1}^{\mathcal{N}} H_k(a) = \frac{n_a}{\mathcal{N}} \frac{1}{\Delta a}, \tag{S14}$$

is a asymptotically unbiased estimator of $f_A(a)$. Since the approximation S11 gets better as Δa gets smaller, smaller *bins* reduce the bias of the estimator.

To guarantee that the estimator is consistent, we also have to verify if its variance goes to zero as $\mathcal{N} \to \infty$. To see this, note that n_a is a sum of i.i.d. Bernoulli random variables (each indicating whether a sample falls in $[a - \Delta a/2, a + \Delta a/2]$). Therefore, the variance of n_a (the sum of the Bernoulli RVs which have variance of $p(1 - p)$) will be the sum of the variances:

$$\left\langle (\delta n_a)^2 \right\rangle = \mathcal{N} p(1 - p) \approx \mathcal{N} f_A(a)\Delta a, \tag{S15}$$

for small Δa. We conclude that the variance of $\overline{H}(a)$ will be:

$$\left\langle \left(\delta \overline{H}(a) \right)^2 \right\rangle = \frac{1}{\mathcal{N} \Delta a} \left\langle (\delta n_a)^2 \right\rangle \approx \frac{f_A(a)}{\mathcal{N} \Delta a}, \tag{S16}$$

which vanish as $\mathcal{N} \Delta a \to \infty$.

Therefore, for the histogram to converge to the true PDF as $\mathcal{N} \to \infty$, we must ensure that $\Delta a \to 0$ and $\mathcal{N} \Delta a \to \infty$, so that both bias and variance vanish. When these conditions are met, by Chebyshev's inequality, convergence in probability is obtained:

$$\frac{n_a}{\mathcal{N}} \frac{1}{\Delta a} \xrightarrow{P} f_A(a). \tag{S17}$$

Kernel Density Estimation[16,17] is a method to estimate the PDF of a random variable which account for smoothness. Instead of counting the number of samples in each bin, the following function is defined:

$$F_h(a) = \frac{1}{h} K \left(\frac{a - A}{h} \right), \tag{S18}$$

where K is the *kernel*, a non-negative function, typically smooth and normalized, and h is a smoothing parameter called *bandwidth*, which can be altered. It is possible to show that the sample average of $\overline{F_h}$ also converges in probabilities to the PDF as $\mathcal{N} \to \infty$,

$$\overline{F_h}(a) = \frac{1}{\mathcal{N} h} \sum_{k=1}^{\mathcal{N}} K\left(\frac{a - A_k}{h}\right) \xrightarrow{P} f_A(a), \tag{S19}$$

under the conditions $h \to 0$ (for bias reduction) and $\mathcal{N} h \to \infty$ (for vanishing variance). Due to mathematical convenience, the normal density function is often employed as a kernel. In this case, the estimator for the PDF becomes:

$$\overline{F_h}(a) = \frac{1}{\mathcal{N} h\sqrt{2\pi}} \sum_{k=1}^{\mathcal{N}} \exp\left(-\frac{(a - A_k)^2}{2h^2}\right). \tag{S20}$$

The bandwidth factor can be optimally selected using a “rule of thumb”.[18] In this work (see main text), the KDE method with Gaussian kernel has been used to estimate the $^1J_{\mathrm{PtPt}}$-coupling and σ-donation distributions by sampling AIMD simulations. The bandwidth was determined according to simple the Scott’s rule[19], as implemented in SciPy Stats module[20]:

$$h = n^{-1/(d+4)}\sigma, \tag{S21}$$

where n is the number of data points and d denotes the number of dimensions (here, $d = 1$) and σ is the standard deviation of the data.

S3 Complementary Material

S3.1 Assessment of the CPMD Simulations

In an ideal NVE simulation, the total energy should remain constant. However, in CPMD, energy drift can occur due to adiabaticity violations—causing energy transfer between ionic and electronic degrees of freedom—or even numerical integration errors (time-step-related inaccuracies). For instance, if the fictitious electron mass μ is too large, the electrons can overheat, leading to energy transfer to ions, leading to a unphysical temperature rise. This breaks the Born-Oppenheimer approximation, making the simulation unreliable. Monitoring temperature fluctuations, therefore, helps detect such energy drifts, indicating whether the simulation is truly microcanonical. Figure S1 illustrates the instantaneous fluctuations and the cumulative average of temperature in the evaluated AIMD simulations, along with the calculated kinetic energy drift ΔE_{kin}. The observed small drift and stable temperature evolution are consistent with well-behaved CPMD simulations reported in the literature[21,22]

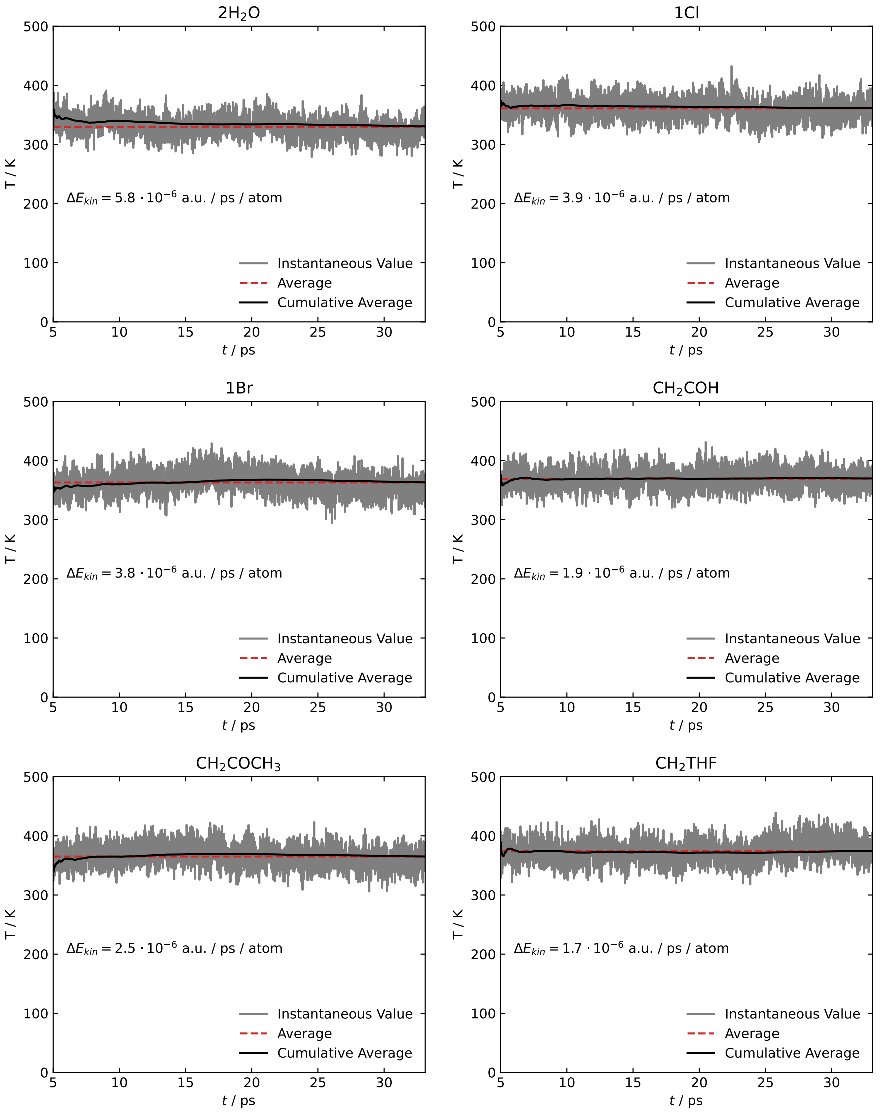

Figure S1: Temperature evolution of the CPMD simulations in the microcanonical ensemble and the fictitious kinetic energy drift ΔE_{kin}.

S3.2 Solvent Structure

AIMD relies on quantum mechanical methods (usually DFT) to describe interatomic forces. It is well-known that many DFT functionals (e.g., GGA-PBE) tend to overstructure liquid water, making hydrogen bonds too strong compared to experiments. Verifying the radial distribution functions (RDFs: O-O, O-H, H-H) against neutron/X-ray scattering experiments ensures the chosen functional correctly captures water's hydrogen-bonding network. In Figure S2, the radial distribution function for water-water interactions is presented. It is possible to see that the simulations reproduce the experimental 295 K liquid water profile.[23] For instance, the simulated O-O RDF shows the first solvation shell peak at $\sim$ 2.8 Å and a second-shell peak at $\sim$ 4.5 Å, while the O-H RDF exhibits a first peak at $\sim$ 1.8 Å (corresponding to hydrogen bond distance)—all in close agreement with experiment. This suggests that the water molecules in our relatively small model system exhibit the expected bulk solvent behavior.

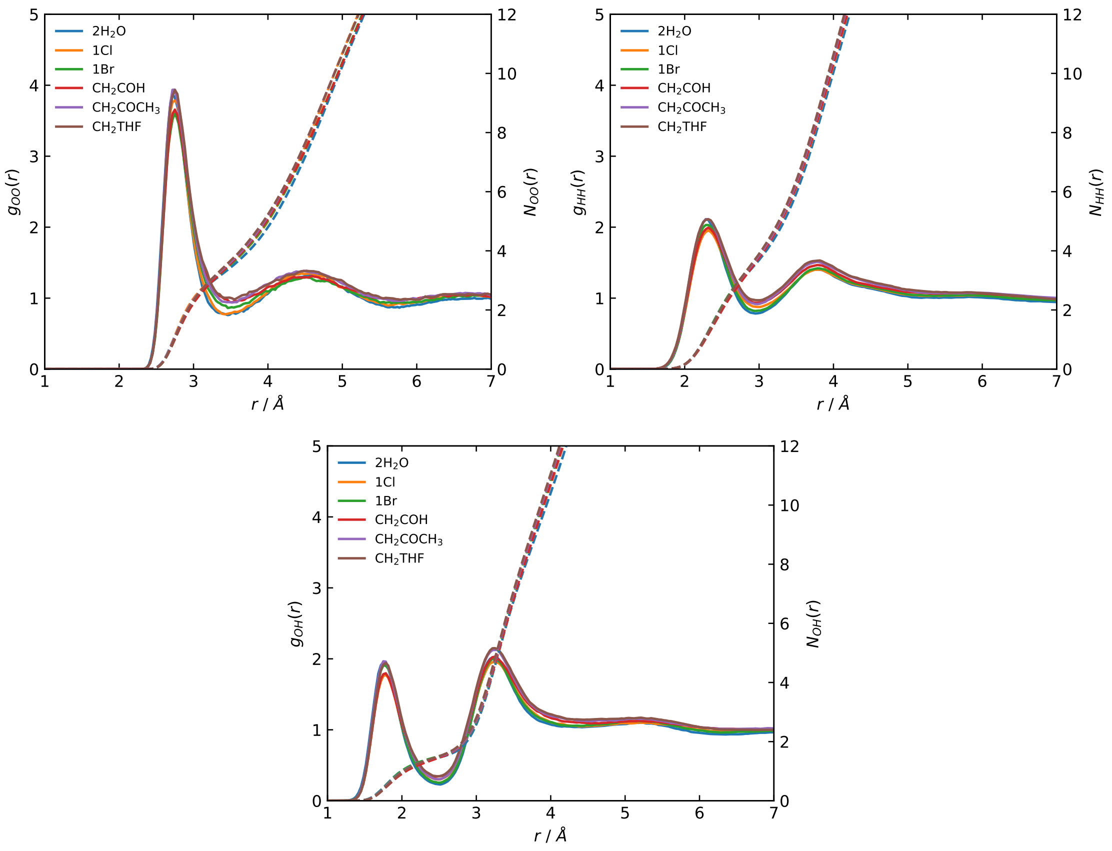

Figure S2: O-O, H-H and O-H RDFs of the solvent's water molecules.

S3.3 Coordination Number Calculation

The coordination number of a specific A atom with respect to X-type atoms can be defined as:[24]

$$\mathrm{CN}_{\mathrm{AX}} = \sum_{i=1}^{N_X} \frac{1-\left(\frac{d_{\mathrm{AX_i}}}{d_0}\right)^p}{1-\left(\frac{d_{\mathrm{AX_i}}}{d_0}\right)^q}, \tag{S22}$$

where N_{X} is the number of X-type atoms, $d_{\mathrm{AX_i}}$ are the $\mathrm{A{-}X_i}$ distances and d_0 is a chosen scale parameter introduced together with the $p, q \in \mathbb{N}$ exponents to ensure a smooth decay of the coordination number.[25] In this study, the average $\mathrm{Pt_1}$ coordination number with respect to oxygen atoms ($\mathrm{CN_{PtO}}$) was employed to quantify the interaction of the $\mathrm{Pt_1}$ center with water molecules at the axial cite. The reference distance d_0 was taken to be 2.6 Å and the curvature parameters, $p = q/2 = 8$.

S3.4 Convergence of $^1J_{\mathrm{PtPt}}$

In Section S2, we saw that the sample average becomes a more precise estimator as the number of samples increases, ultimately converging to the ensemble average. Figure S3 illustrates the evolution of $^1J_{\mathrm{PtPt}}$ across all evaluated simulations. The results indicates that convergence is successfully achieved with 256 sampled configurations. As discussed in the main text, the fluctuations in $^1J_{\mathrm{PtPt}}$ visibly decrease as the σ-donation of the ligand increases—compare, for example, the fluctuations in **$2H_2O$** with those in **CH_2THF**.

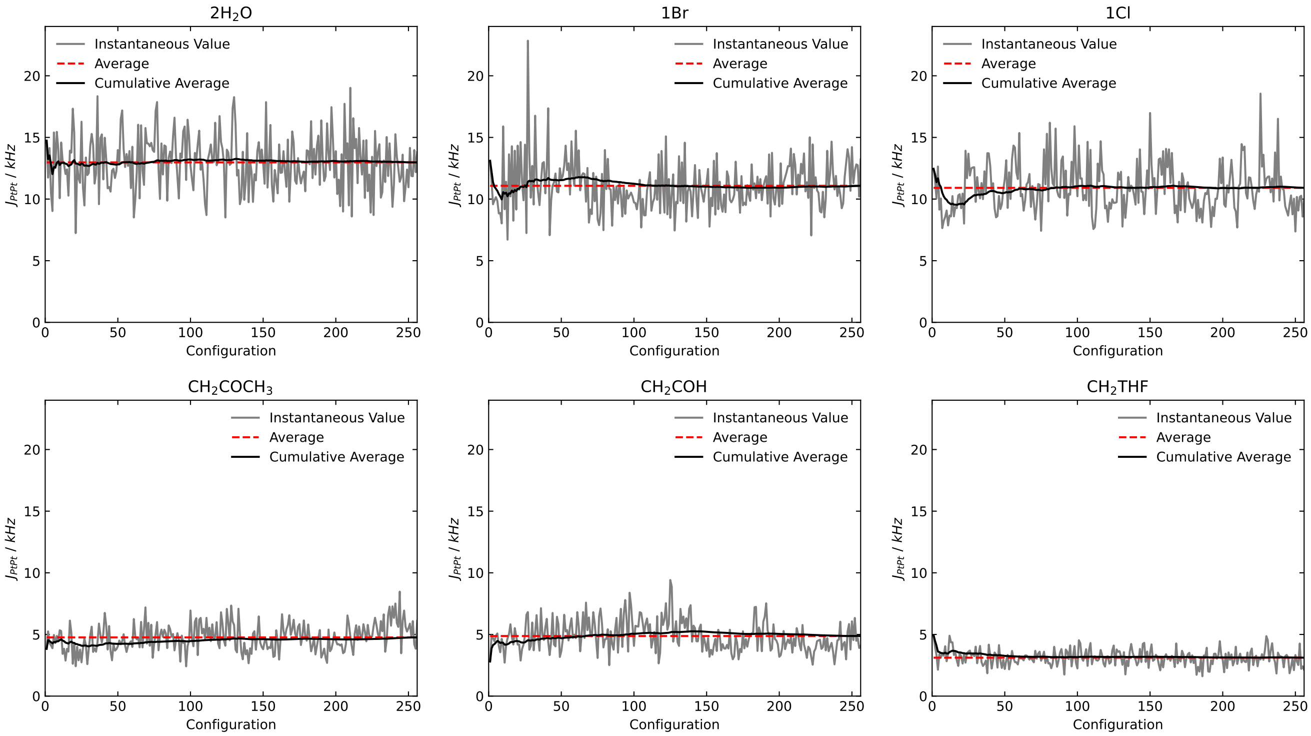

Figure S3: Temporal evolution of ${}^{1}J_{\mathrm{PtPt}}$ across sampled configurations from AIMD simulations. The sampling interval, total simulation time, time discretization, and other simulation parameters are detailed in the Computational Methods in the main text.

S3.5 Convergence of σ-Donation

Figure S4 shows the convergence of the extent of σ-donation across sampled simulation configurations. The cumulative average smoothly converges to a stable value for all simulations, indicating 64 configurations are sufficient for reliable statistical sampling of this property. Note that the **1Cl** complex system exhibits two distinct bonding regimes. Figure S6 presents the correlation between σ-donation and the d_{ClPt_2} distance, along with conditional expectation estimate of the Cl$-$Pt$_2$ bond distance. Note how this estimate differs significantly between the two regimes, reflecting their distinct electronic structures.

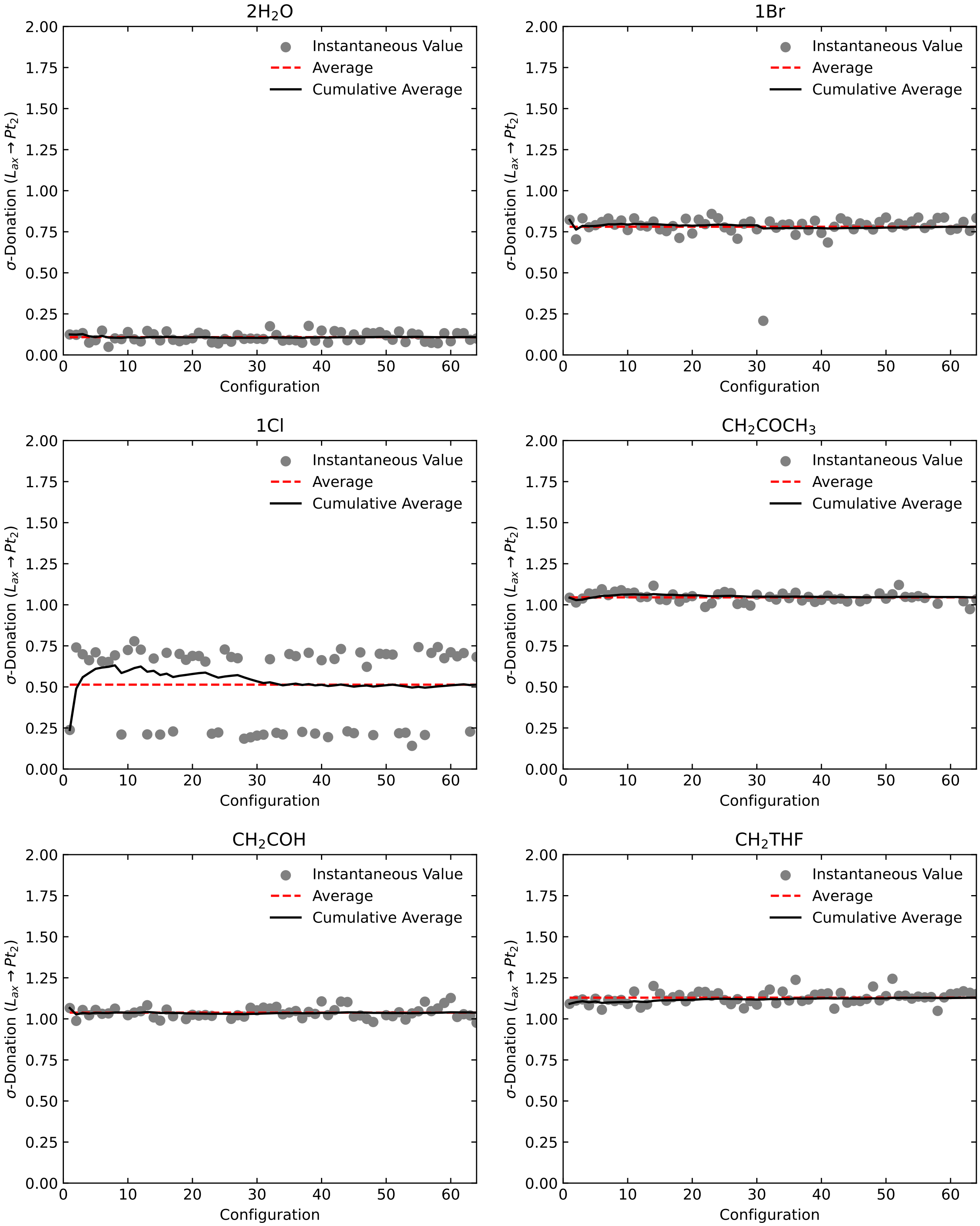

Figure S4: Evolution of σ-donation computed from sampling configurations of the AIMD simulations.

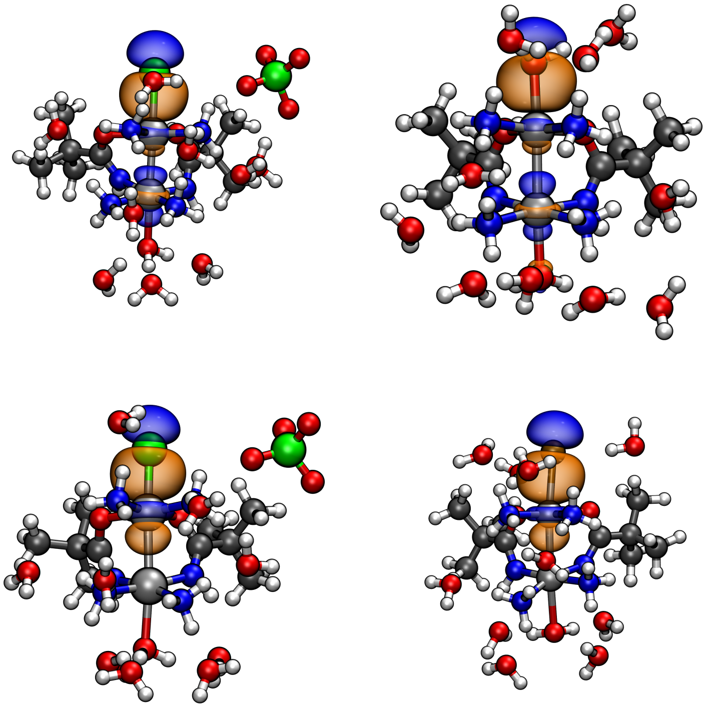

Figure S5: σ(L-Pt) NLMOs of the **1Cl** complex compared to weaker (**2H$_2$O**) and stronger (**1Br**) σ-donors. The NLMO of the lone-pair-dominant regime (top left) closely resembles that of the **2H$_2$O** complex (top right), while the bonding-enhanced regime (bottom left) aligns more closely with the **1Br** complex (second row right).

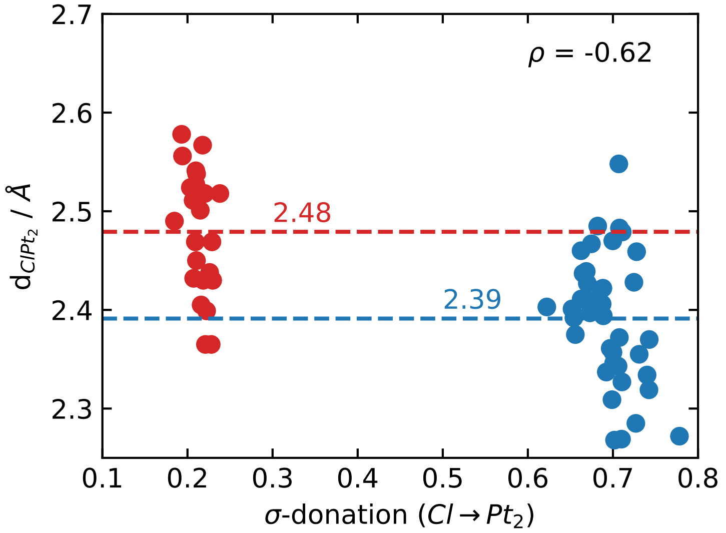

Figure S6: Sampled configurations mapped to σ-donation $\times$ $d_{\mathrm{ClPt_2}}$ space. A spearman correlation coefficient of -0.64 indicates a strong negative correlation.

The data presented in Figure 7 (right; long Pt-Pt at very high $^1J_{\mathrm{PtPt}}$) and in Figure S6 (short Pt-Cl at low σ-donation) show scattered points that appear to be outliers when visually analyzed. However, it should be noted that this observed range is associated with the fluctuations of the Pt-Pt and Pt-Cl bonds in

solution. This demonstrates the importance of dynamically treating these compounds to better understand their behavior and NMR properties in solution, as these variations have the potential to influence the total averaged property, as before reported for similar complexes.[26] Furthermore to guarantee that we are working with significant data points, we performed the Grubbs' statistical test,[27] whose results are shown in the Figure S7. For data presented in Figure 7, no outliers were identified and in Figure S6, only one was identified as outlier, which was then removed with no impact in the σ-donation study.

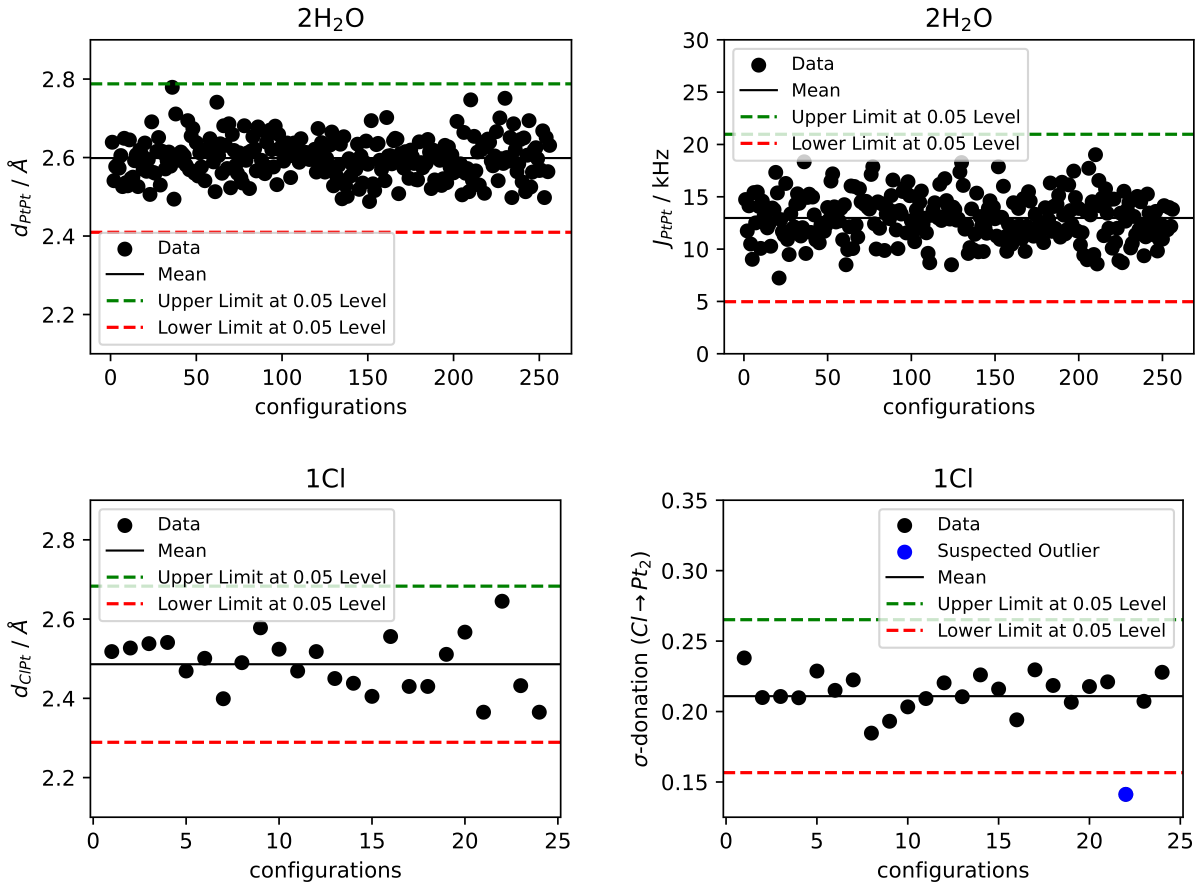

Figure S7: Grubbs' statistical test for outliers for the Pt-Pt bond lenght and $^1J_{\mathrm{PtPt}}$ data from Figure 7 and for Cl-Pt bond lenght and σ-donation data from Figure S6 for short Pt-Cl at low σ-donation.

S3.6 π-donation and π-backdonation

We also performed an analysis to address the role of π-interactions related to the halogen ligands p-orbitals. The quantification of π-donation and π-back-donation was achieved by measuring the metal contribution to the halide lone-pair NLMOs, and the delocalization of nonbonding t_{2g} metal d orbital to the halide axial ligand, respectively. The analysis (shown in Figure S8) indicates that both π-donation and π-back-donation are weak, with halide ligand atomic orbitals contributing over 99% of the density of the lone-pair NLMOs. This observation further supports the conclusion that the observed bonding is primarily characterized by σ-donation with an negligible π component.

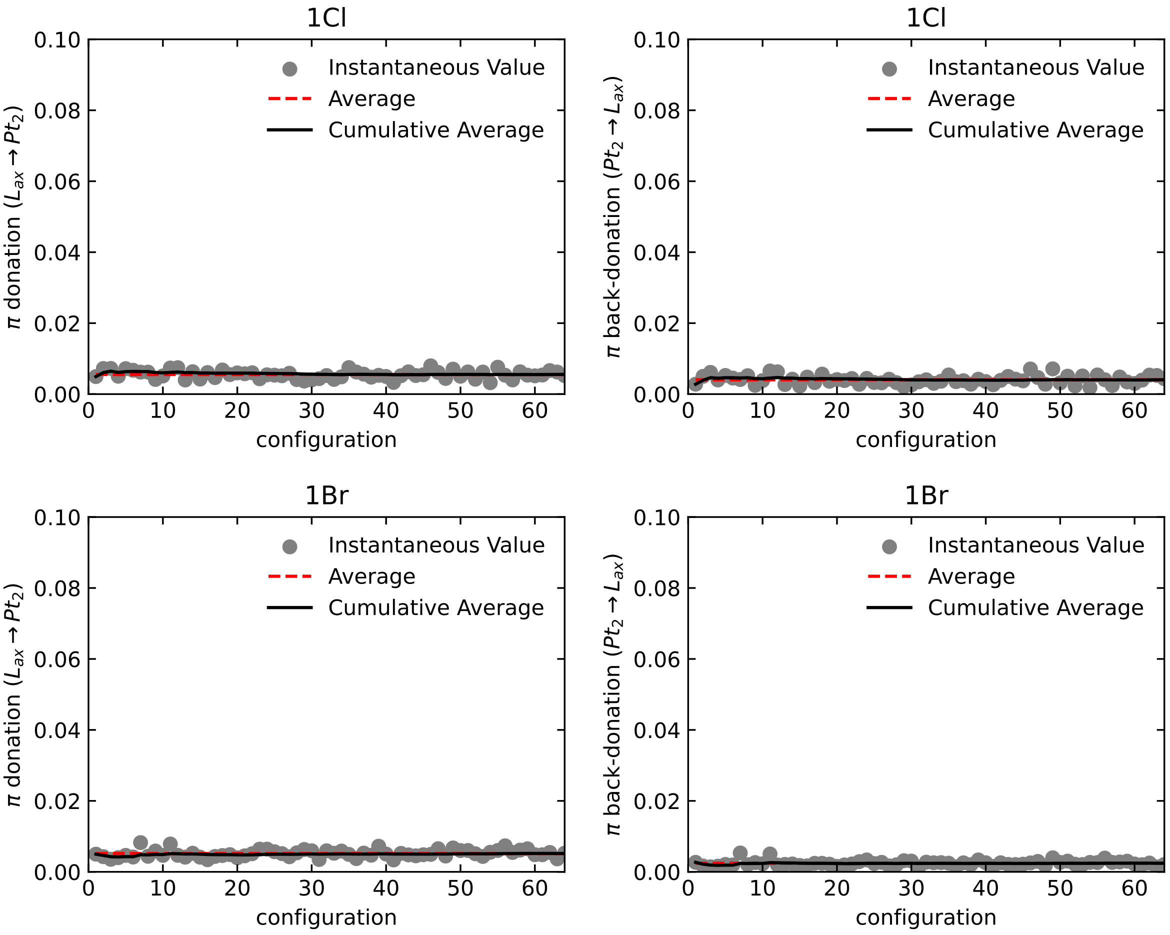

Figure S8: Evolution of π-donation of the *p*-orbitals of Cl and Br ligands and and π-back-donation of the Pt t_{2g} d orbital computed from sampling configurations of the AIMD simulations.

S3.7 NLMO Decomposition of $^1J_{\mathrm{PtPt}}$

Figure S9 presents the decomposition of the $^1J_{\mathrm{PtPt}}$-coupling in the NLMO basis (with orbital labels directly obtained from the ADF package calculations). Note how the primary contributing orbital – the Pt−Pt orbital – exhibits a significant reduction in its contribution, both in absolute and relative magnitudes.

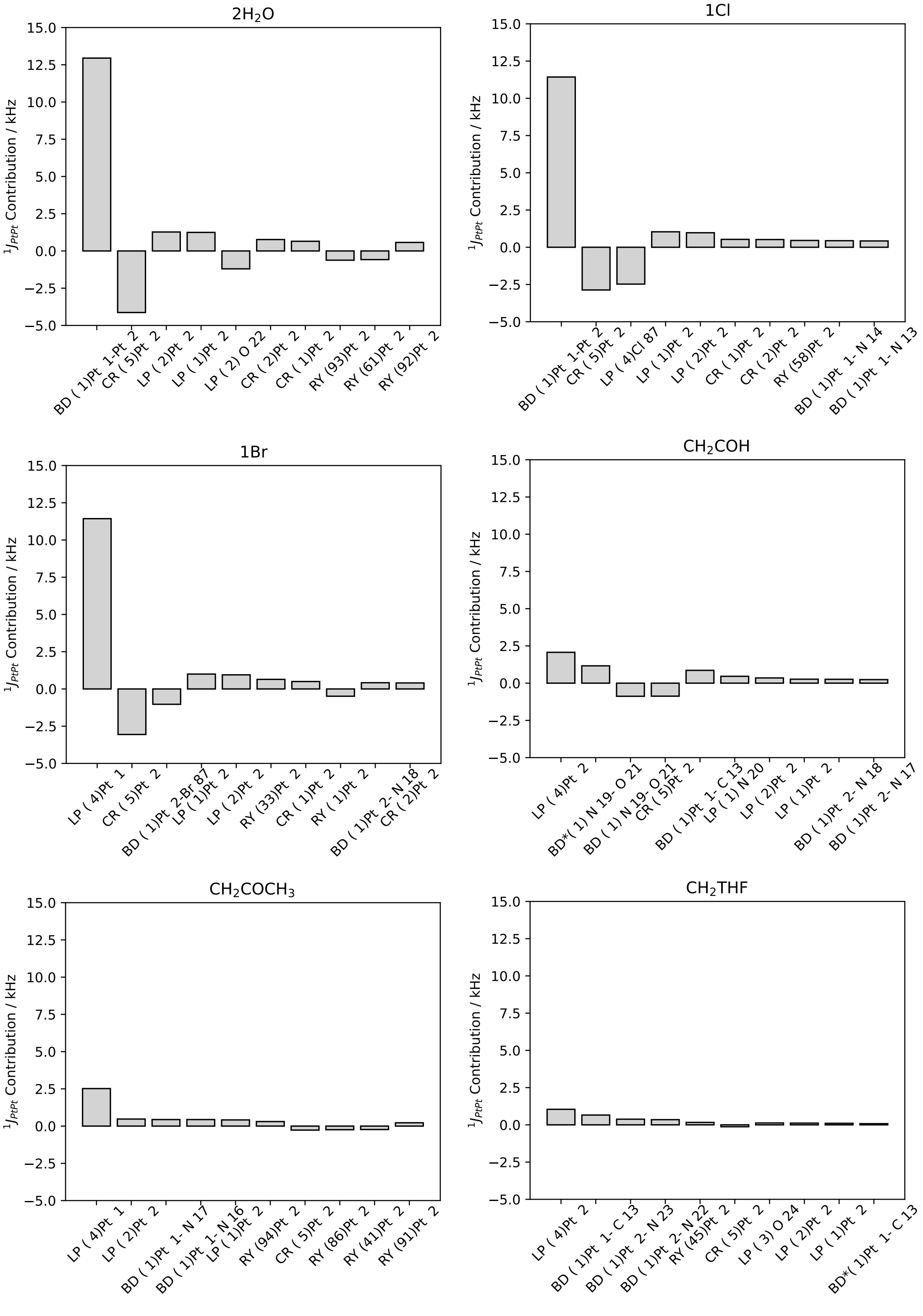

Figure S9: ${}^{1}J_{\mathrm{PtPt}}$ NLMO decomposition into its main contributing orbitals.

References

(1) Martínez, L.; Andrade, R.; Birgin, E. G.; Martínez, J. M. PACKMOL: A package for building initial configurations for molecular dynamics simulations. *J. Comput. Chem.* **2009**, *30*, 2157–2164.

(2) Grimme, S. Accurate description of van der Waals complexes by density functional theory including empirical corrections. *J. Comput. Chem.* **2004**, *25*, 1463–1473.

(3) Nosé, S. A unified formulation of the constant temperature molecular dynamics methods. *J. Chem. Phys.* **1984**, *81*, 511–519.

(4) DiStasio Jr., R. A.; Santra, B.; Li, Z.; Wu, X.; Car, R. The individual and collective effects of exact exchange and dispersion interactions on the ab initio structure of liquid water. *J. Chem. Phys.* **2014**, *141*, 84502.

(5) Brehm, M.; Kirchner, B. TRAVIS—A Free Analyzer and Visualizer for Monte Carlo and Molecular Dynamics Trajectories. *J. Chem. Inf. Model.* **2011**, *51*, 2007–2023.

(6) Brehm, M.; Thomas, M.; Gehrke, S.; Kirchner, B. TRAVIS-A free analyzer for trajectories from molecular simulation. *J. Chem. Phys.* **2020**, *152*, 164105.

(7) Humphrey, W.; Dalke, A.; Schulten, K. VMD – Visual Molecular Dynamics. *J. Mol. Graph.* **1996**, *14*, 33–38.

(8) Batista, P. R.; Ducati, L. C.; Autschbach, J. Solvent Effect on the ^{195}Pt NMR Properties in Pyridonate-Bridged Pt^{III} Dinuclear Complex Derivatives by ab initio Molecular Dynamics and Localized Orbital Analysis. *Phys. Chem. Chem. Phys.* **2021**, *23*, 12864–12880.

(9) Köddermann, T.; Paschek, D.; Ludwig, R. Molecular dynamic simulations of ionic liquids: A reliable description of structure, thermodynamics and dynamics. *ChemPhysChem* **2007**, *8*, 2464–2470.

(10) Glendening, E. D.; Landis, C. R.; Weinhold, F. NBO 6.0: Natural bond orbital analysis program. *J. Comput. Chem.* **2013**, *34*, 1429–1437.

(11) Adamo, C.; Barone, V. Toward reliable density functional methods without adjustable parameters: The PBE0 model. *J. Chem. Phys.* **1999**, *110*, 6158–6170.

(12) Moncho, S.; Autschbach, J. Relativistic Zeroth-Order Regular Approximation Combined with Nonhybrid and Hybrid Density Functional Theory: Performance for NMR Indirect Nuclear Spin-Spin Coupling in Heavy Metal Compounds. *J. Chem. Theory Comput.* **2010**, *6*, 223–234.

(13) Franchini, M.; Philipsen, P. H. T.; Visscher, L. The Becke Fuzzy Cells Integration Scheme in the Amsterdam Density Functional Program Suite. *J. Comput. Chem.* **2013**, *34*, 1819–1827.

(14) Tuckerman, M. E. *Statistical Mechanics: Theory and Molecular Simulation;* Oxford University Press: New York, 2023.

(15) Papoulis, A. *Probability, Random Variables, and Stochastic Processes;* McGraw-Hill: New York, 3 ed.; 1991.

(16) Parzen, E. On estimation of a probability density function and mode. *Ann. Math. Stat.* **1962**, *33*, 1065–1076.

(17) Rosenblatt, M. Remarks on some nonparametric estimates of a density function. *Ann. Math. Stat.* **1956**, *27*, 832–837.

(18) Turlach, B. A. "Bandwidth selection in kernel density estimation: a rewiew", Technical Report, Humboldt Universitaet Berlin, 1993.

(19) Scott, D. W. *Multivariate density estimation: theory, practice, and visualization;* John Wiley & Sons: New York, USA, 2015.

(20) Virtanen, P.; Gommers, R.; Oliphant, T. E.; Haberland, M.; Reddy, T.; Cournapeau, D.; Burovski, E.; Peterson, P.; Weckesser, W.; Bright, J.; van der Walt, S. J.; Brett, M.; Wilson, J.; Millman, K. J.; Mayorov, N.; Nelson, A. R. J.; Jones, E.; Kern, R.; Larson, E.; Carey, C. J.; Polat, İ.; Feng, Y.; Moore, E. W.; VanderPlas, J.; Laxalde, D.; Perktold, J.; Cimrman, R.; Henriksen, I.; Quintero, E. A.; Harris, C. R.; Archibald, A. M.; Ribeiro, A. H.; Pedregosa, F.; van Mulbregt, P.; SciPy 1.0 Contributors, SciPy 1.0: Fundamental Algorithms for Scientific Computing in Python. *Nat. Methods* **2020**, *17*, 261–272.

(21) Ducati, L. C.; Marchenko, A.; Autschbach, J. NMR J-coupling constants of Tl–Pt bonded metal complexes in aqueous solution: Ab-initio molecular dynamics and localized orbital analysis. *Inorg. Chem.* **2016**, *55*, 12011–12023.

(22) VandeVondele, J.; Krack, M.; Mohamed, F.; Parrinello, M.; Chassaing, T.; Hutter, J. Quickstep: Fast and accurate density functional calculations using a mixed Gaussian and plane waves approach. *Comput. Phys. Commun.* **2005**, *167*, 103–128.

(23) Soper, A. K. Water and ice structure in the range 220–365 K from radiation total scattering experiments. *Water: Fundamentals as the Basis for Understanding the Environment and Promoting Technology* **2014**, 151–171.

(24) Lau, J. K.-C.; Ensing, B. Hydrolysis of cisplatin—a first-principles metadynamics study. *Phys. Chem. Chem. Phys.* **2010**, *12*, 10348–10355.

(25) Iannuzzi, M.; Laio, A.; Parrinello, M. Efficient Exploration of Reactive Potential Energy Surfaces using Car-Parrinello Molecular Dynamics. *Phys. Rev. Lett.* **2003**, *90*, 238302.

(26) Batista, P. R.; Ducati, L. C.; Autschbach, J. Dynamic and relativistic effects on Pt-Pt indirect spin-spin coupling in aqueous solution studied by ab initio molecular dynamics and two- vs four-component density functional NMR calculations. *J. Chem. Phys.* **2024**, *160*, 114307.

(27) Grubbs, F. Sample Criteria for Testing outlying observations in samples. *Ann. Math. Stat.* **1950**, *21*, 27–28.
